# A deep inverse convolutional neural network-based semantic classification method for land cover remote sensing images

**DOI:** 10.1038/s41598-024-57408-0

**Published:** 2024-03-27

**Authors:** Ming Wang, Anqi She, Hao Chang, Feifei Cheng, Heming Yang

**Affiliations:** 1 Network Information Center, Jilin Normal University, Siping, 136000 China; 2 Human Resource Department, Jilin Normal University, Siping, 136000 China; 3 Jilin Normal University Affiliated Experimental School, Siping, 136000 China; 4 School of Geographic Science and Tourism, Jilin Normal University, Siping, 136000 China

**Keywords:** Deep inverse convolutional neural network, Land cover, Remote sensing images, Semantic classification, Semantic segmentation, Feature extraction, Ecology, Climate sciences, Ecology, Environmental sciences, Environmental social sciences, Engineering

## Abstract

The imbalance of land cover categories is a common problem. Some categories appear less frequently in the image, while others may occupy the vast majority of the proportion. This imbalance can lead the classifier to tend to predict categories with higher frequency of occurrence, while the recognition effect on minority categories is poor. In view of the difficulty of land cover remote sensing image multi-target semantic classification, a semantic classification method of land cover remote sensing image based on depth deconvolution neural network is proposed. In this method, the land cover remote sensing image semantic segmentation algorithm based on depth deconvolution neural network is used to segment the land cover remote sensing image with multi-target semantic segmentation; Four semantic features of color, texture, shape and size in land cover remote sensing image are extracted by using the semantic feature extraction method of remote sensing image based on improved sequential clustering algorithm; The classification and recognition method of remote sensing image semantic features based on random forest algorithm is adopted to classify and identify four semantic feature types of land cover remote sensing image, and realize the semantic classification of land cover remote sensing image. The experimental results show that after this method classifies the multi-target semantic types of land cover remote sensing images, the average values of Dice similarity coefficient and Hausdorff distance are 0.9877 and 0.9911 respectively, which can accurately classify the multi-target semantic types of land cover remote sensing images.

## Introduction

Remote sensing technology is a comprehensive detection technology that rose in the 1960s^1,2^. Various sensors are used to detect the radiation or reflected electromagnetic wave information of surface objects from a long distance, and the detected electromagnetic wave information is processed and synthesized into image data, so as to realize the recognition and classification of actual surface objects^3,4^. With the rapid development of remote sensing technology towards high spatial resolution, hyperspectral resolution and high temporal resolution, people can obtain more and more large-scale remote sensing image data^5,6^. In recent decades, with the diversification and diversified development of sensor platforms and the substantial improvement of remote sensing image spatial and spectral resolution, as well as the continuous development of pattern recognition technology, computer automatic control technology, GIS system and cognitive system technology^7^, new theories and methods of remote sensing digital image processing are constantly emerging in remote sensing image computer interpretation In terms of visual interpretation and human–computer interaction processing of remote sensing images, the classification technology of remote sensing images has been constantly improved^8^. Many researchers have extended pattern recognition methods to land cover remote sensing image data, and conducted a lot of useful research in feature extraction, improving classification accuracy, and innovation of classification technology^9^. Image semantic classification refers to the process of automatic recognition and classification of semantic information such as objects, scenes or emotions in images^10^. It understands the essential meaning of the image by analyzing the visual features, context information and human language description in the image, and classifies the multi-target information in the image^11^. The purpose of image semantic classification is to enable computers to “understand” image content and distinguish different objects, scenes or emotions^12^.

In the research of remote sensing image classification and recognition, there are a lot of references. Literature^13^ Ozyurt has proposed a remote sensing image recognition method based on convolutional neural network. This method uses convolutional neural network to extract remote sensing image features in an efficient depth for image recognition. Although it has achieved good remote sensing image classification results, its classification ability is only limited to image type classification, The ability of semantic classification within images needs further testing. Literature^8^ Matsunobu et al. studied the cloud detection method of remote sensing image based on convolutional neural network. This method uses convolution operation to realize local perception of remote sensing image, and extracts local features by sliding filters on remote sensing image, thus completing accurate detection of remote sensing image. However, the cloud detection method of remote sensing image based on convolutional neural network also has some shortcomings, For example, the training process is tedious and the convergence effect is poor. Literature^14^ Samaneh et al. proposed a remote sensing image target segmentation method based on the gated residual supervision network. The gated residual supervision network has a strong supervisory learning ability, which can be trained through tag data to learn the feature representation of remote sensing image target objects. This method can make full use of the prior information and tag data in remote sensing images, improve the accuracy of target segmentation, and facilitate remote sensing image classification and recognition. However, this method needs a lot of computing resources and time to infer. For large-scale remote sensing image data sets, its application is limited, and its application performance is limited by image segmentation. Further research is needed in the semantic classification and recognition of multiple objects in the image area. Reference^15^ Yoshida et al. used a deep learning based method to classify land cover in aerial photography images of the Xuchuan River, improving classification accuracy by connecting data related to the model output. Use the modified deep learning model to segment aerial photography images and classify different types of land cover. The model trained by this method in specific regions or scenarios may not have good generalization ability, and the classification effect on data from other regions or under different conditions may not be ideal. Reference^16^ Kavran et al. proposed an object based spatiotemporal method for satellite image classification using graph neural networks. Construct a directed graph by connecting segmented land regions, then use convolutional neural networks to extract features, and use graph neural networks for node classification. This method may have certain limitations on the effectiveness of land cover classification in different regions or scenarios, and has significant computational and storage requirements.

The gap pointed out in previous research mainly focuses on the semantic classification and recognition of remote sensing images. Although some achievements have been made in the classification of image types, there are still many challenges in accurately segmenting and classifying semantic information within images, such as target objects. For example, traditional algorithms are prone to problems of over segmentation and under segmentation, resulting in low semantic segmentation accuracy. In addition, feature extraction methods usually only consider a single or limited set of features and fail to fully capture the comprehensive features of different targets in the image. Finally, there is still significant room for improvement in utilizing the interrelationships between features in classification and recognition methods to improve classification accuracy. Therefore, this paper proposes a semantic classification method for land cover remote sensing images based on deep deconvolution neural networks. Compared with previous research, the innovation and contribution of this paper are mainly reflected in the following aspects:

1. The use of deep deconvolution neural networks for multi-target semantic segmentation of land cover remote sensing images has improved the accuracy and precision of classification.
2. Introducing an improved sequential clustering algorithm for object segmentation solves the problems of over segmentation and under segmentation in traditional algorithms, further improving the semantic segmentation accuracy of land cover remote sensing images.
3. In the feature extraction stage, a semantic feature extraction method that comprehensively considers color, texture, shape, and size is adopted to comprehensively capture the feature information of different targets in land cover remote sensing images.
4. The application of a semantic feature classification and recognition method based on random forest algorithm fully utilizes the interrelationships between features, significantly improving the semantic classification accuracy of land cover remote sensing images.

In summary, this paper proposes an innovative and practical semantic classification method for land cover remote sensing images through in-depth research and improvement, which has contributed to the development of remote sensing image classification and recognition. However, although the method proposed in this paper has been improved and broken through in multiple aspects, there are still some limitations. For example, for remote sensing images in complex backgrounds, the method proposed in this paper may not be able to completely eliminate interference factors, resulting in a decrease in classification accuracy. In addition, for large-scale remote sensing image data, the method proposed in this paper may have performance bottlenecks in real-time processing.

## Semantic classification of land cover remote sensing images

### Semantic segmentation algorithm for land cover remote sensing images based on deep inverse convolutional neural network

The complexity and diversity of land cover types make it difficult to label the dataset, which in turn leads to ambiguity and error in the segmentation results. Therefore, a deep inverse convolutional neural network is used for semantic segmentation of land cover remote sensing images. Compared with the traditional convolutional neural network, the deep inverse convolutional network can convert low-resolution feature maps into high-resolution prediction maps, improve the accuracy and detail expression ability of semantic segmentation, understand and describe different land cover types in remote sensing images in a more comprehensive way, and provide more accurate inputs for the subsequent classification tasks.

#### Semantic segmentation network architecture for land cover remote sensing images

The general deep convolutional network will reduce the dimension size of the feature layer layer by layer, and the input image size is much smaller than the output image. For the task of semantic segmentation of land cover remote sensing images, every pixel of the image is an object to be segmented, and the previous convolutional network structure will no longer be applicable^17^.

For the task of semantic segmentation of land cover remote sensing images based on deep inverse convolutional neural network, the usual method to solve this problem is to divide the land cover remote sensing images into a number of small blocks in advance, and then according to whether or not the pixel in the center of the image block belongs to the target organization, a semantic label is given to the image block, which is inputted to the network together with the image block to realize the task of single-label classification of the semantics of land cover remote sensing images. This method increases the complexity of image preprocessing, and the whole network needs to preprocess a large number of data blocks, which increases the time consumption of computation.

In the past few years, researchers have designed a full convolutional neural network, which is mainly used to achieve semantic segmentation of natural images. This algorithm replaces the last full connection layer in the convolutional network with the convolutional layer, and applies the up sampling and feature layer clipping operations to solve the problem of the inconsistency between the size of the input image and the size of the output image, and realize the pixel wise prediction of the image. Since then, a series of semantic image segmentation algorithms based on convolutional neural network training have been proposed, and the precision of semantic image segmentation has been repeatedly refreshed. DeconvNet is an extension of FCN application. It learns a multi-level deconvolution network, reconstructs the target details of land cover remote sensing image, and effectively solves the problems of easily misdividing small targets and losing target edge details in FCN.

**Figure 1:**
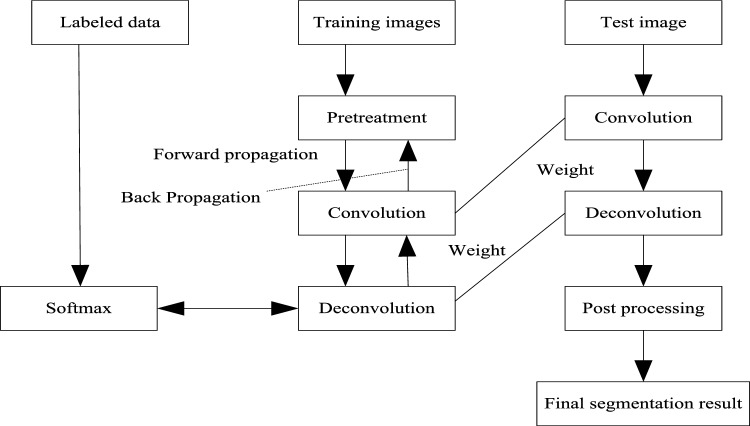
Schematic diagram of supervised learning stage.

Inspired by the latest deep learning algorithms such as FCN and DeconvNet, the semantic segmentation of land cover remote sensing image is completed by using full convolution network and deconvolution technology. The deep deconvolution network used in this paper is a supervised learning method, which includes training stage and testing stage. The details of the learning method block diagram are shown in Fig. 1.

In the training phase, the ten fold cross validation method is used to input the remote sensing image of land cover and the segmented image as training samples into the depth deconvolution neural network. Through forward and backward propagation, the network weights are iteratively trained, and validation samples are set to provide supervision and guidance for training. Finally, a Softmax classifier was trained, and the target loss function was optimized to obtain the probability map of semantic segmentation of the whole land cover remote sensing image^18^; In the test phase, the test image is input into the trained network, and the final test image segmentation result is obtained after a forward propagation calculation.

**Figure 2:**
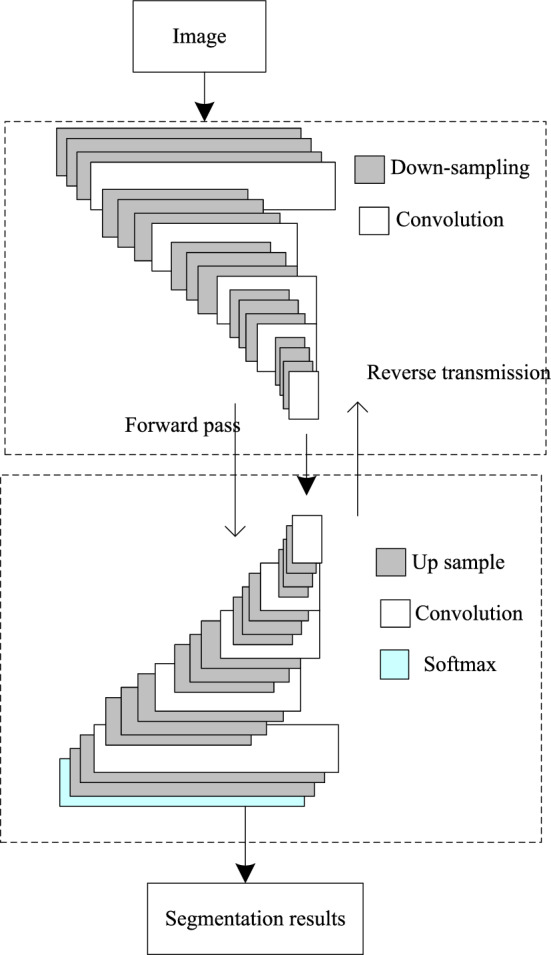
Land cover remote sensing image segmentation algorithm based on deep deconvolution neural network.

The network structure proposed in this paper is shown in Fig. 2. To facilitate network input, the input image is cut to 3 × 224 × 224 pixel size. In the convolutional network part, the structure similar to FCN is adopted, and the final full connection layer is replaced by the convolutional layer. The convolution network consists of five stacked convolution layers, five maximum pooling layers and two complete convolution layers. The convolution adopts the stacking form, that is, one or two consecutive identical convolution layers are set after each convolution layer. The size of convolution kernel in the network is 3 × 3, the step size is 1, and the size of the feature map before and after the convolution operation is consistent by adding a value “0” with a width of 1 at the edge of the input feature map. Stacking convolution layers can not only increase the depth of the network to learn more network parameters, but also effectively avoid over fitting.

The inverse convolutional network part adopts the mirror structure of convolutional network, which aims at reconstructing the shape of the input target, so the multilevel inverse convolutional structure is also able to capture the shape details of different levels of the land-covered remote sensing images like convolutional network. In the convolutional network, the low-level features can describe the whole target rough information, such as target location, general shape, etc., while the more complex high-level features have classification characteristics and contain more target details^19^.

**Figure 3:**
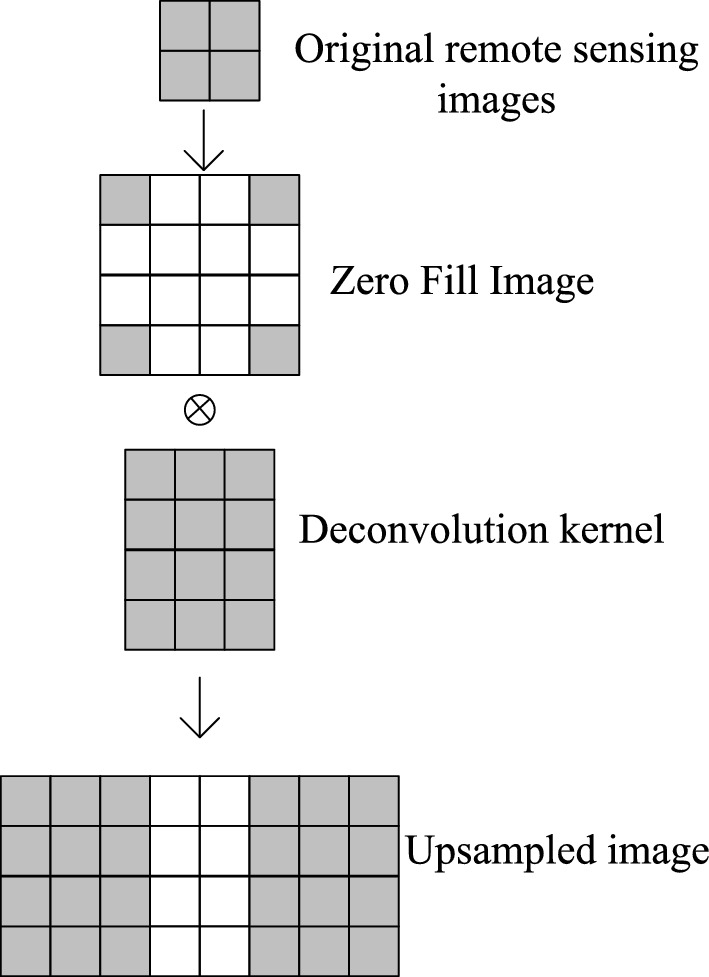
Upsampling structure.

Among them, the up-sampling structure is shown in Fig. 3.

Land cover remote sensing images are up-sampled by zero-filling and deconvolution:

1 $$G_{1} \left( X \right) = \max \left( {V_{1} \times X + A_{1} ,0} \right) + \beta_{i} \min \left( {0,V_{1} \times X + A_{1} } \right)$$

Among them, $X$ denotes the input land cover remote sensing image, parameter $V_{1}$, $A_{1}$ are the up-sampled inverse convolution kernel (weight matrix) and bias. $G_{1} \left( X \right)$ denotes the upsampledimage, the $\beta_{i}$ denotes the correction factor. Here, the inverse convolution can be regarded as the inverse operation of convolution, and the step size is set to be $h$.

The task of semantic segmentation of land cover remote sensing images is actually to predict the category of each pixel, which requires both better characterization of the global features of the target and more preservation of the edge features of the target, and thus has a high demand for detailed features. Different sources of data provide redundant but complementary information, and deep networks are beneficial to extract more information from different data, which provides the possibility to improve the performance of segmentation task through fusion and complementarity. The remote sensing image features are extracted from different data by two neural network branches, and then different region category probability prediction maps are obtained, which correspond to the category prediction results obtained from different remote sensing image target data, where the decision-level fusion is performed, and the results of the two network branches are weighted and fused to further improve the segmentation performance. Using $Y_{1}$, $Y_{2}$ denote the category probability maps of the outputs of different branch networks, respectively. The output of fusion is the result of semantic segmentation of remote sensing images, which is denoted as:

2 $$O_{j} = G_{1} \left( X \right)\left( {\varpi_{1} Y_{1j} + \varpi_{2} Y_{2j} } \right)$$

Among them, $j$ denotes the category number for semantic segmentation of land cover remote sensing images, the $\varpi_{1}$ and $\varpi_{2}$ represent the weighted coefficients, respectively.

The probability maps obtained from different branch networks are fused at the decision level using weighted fusion, and the fused resultant probability maps are obtained, which represent the final probability of the region to which each pixel belongs, respectively. The new probability maps are used to make a category decision based on the maximum probability, e.g., pixels belonging to the target 1 are labeled as 1, and the target 2 pixels are labeled as 0.

#### Deep inverse convolutional neural network training

In order to ensure the accuracy of remote sensing image segmentation, the multi-loss objective function is established as follows:

3 $$Z_{s} = \left( {1 - Z_{G} } \right)Z_{c} + Z_{G} + \left( {1 - 50^*Z_{G} } \right)Z_{a} O_{j} + 50^*Z_{G} + \alpha_{e} Z_{e}$$

In the formula, the $Z_{s}$, $Z_{c}$ are the total objective loss function, the input remote sensing image and the cross-entropy loss of the source domain labeling; the $Z_{a}$, $Z_{e}$ are adversarial loss, cross-entropy loss from different images (source and target images); the $Z_{G}$ is the learning rate corresponding to the segmentation network; the $\alpha_{e}$ is a fixed balance coefficient used to control the difference balance from different remote sensing image samples.

For the input land cover remote sensing image from the labeled source domain dataset as $X_{m}$, mark the corresponding one pot code as $Y_{m}$, for which the network predicts the results as $O\left( {X_{m} } \right)$, the corresponding cross-entropy loss is as follows:

4 $$Z_{ce} = Z_{s} \sum\limits_{k,u} {\sum\limits_{b \in B} {Y_{m}^{{\left( {k,u,b} \right)}} } } \lg \left( {O\left( {X_{m} } \right)^{{\left( {k,u,b} \right)}} } \right)$$

Among them, $k$, $u$, $b$ are the three-channel size of theinput land cover remote sensing images $X_{m}$, respectively.

In the training process, a discriminant network is designed, which uses the SegNet network, use $F\left( \cdot \right)$ to denote, and the adversarial loss based on this network is:

5 $$Z_{a} = - \sum\limits_{k,u} {\lg \left( {F\left( {O\left( {X_{m} } \right)^{{\left( {k,u} \right)}} } \right)} \right)}$$

The purpose of this adversarial loss is to make the predicted results of semantic segmentation of land cover remote sensing images closer and closer to the labeling of the source domain.

Adversarial training using unlabeled samples of land cover remote sensing images in a semi-supervised environment. For unlabeled target data, the adversarial loss that $Z_{a}$ is still usable, but it cannot be used because there is no labeling information. In addition, the performance of the network is degraded when applying only the adversarial loss to unlabeled target data, because the discriminant network has regularization to correct the prediction, and correcting it only with the adversarial loss will make the segmentation prediction overfitting the source domain annotation. Therefore, a “self-learning” strategy is adopted, which is able to train the discriminator using unlabeled target data^20^. The main idea is that training the discriminator generates a confidence map, i.e., the $F\left( {O\left( {X_{m} } \right)^{{\left( {k,u} \right)}} } \right)$, it can find the region where the distribution between the prediction result and the source domain annotation is close enough, and then binarize the segmented prediction confidence map with the corresponding confidence map of the source domain annotation, and use a threshold to determine their correlation and thus find the confidence region. Namely $\hat{X}_{m} = \arg \max \left( {O\left( {X_{m} } \right)} \right)$. The constructed semi-supervised losses are as follows:

6 $$Z_{s} = - \sum\limits_{k,u} {\sum\limits_{b \in B} {I\left( {F\left( {O\left( {X_{m} } \right)^{{\left( {k,u} \right)}} } \right) > H_{s} } \right)\sum {\hat{X}_{m} \lg O\left( {X_{m} } \right)^{{\left( {k,u,b} \right)}} } } }$$

Among them, $I$ refers to the indexing function; the $H_{s}$ denotes the threshold parameter for unlabeled target data. During the training process, the self-learning target, the $\hat{X}_{m}$ and index function values are assumed to be constants, so Eq. (6) can be considered as the global cross-entropy loss based on the target features. Several experiments have shown that the threshold value $H_{s}$ its effect is better in the interval of 0.1–0.3. After the network training, the semantic segmentation of remote sensing images is completed using Eq. (2).

### Semantic feature extraction method for remote sensing images based on improved sequential clustering algorithm

In the articles on image recognition and retrieval, most of the meanings expressed by the term image semantics refer to the terminology of how to utilize the information of an image, especially the high-level information, to provide a way of describing the image for research. Therefore, image semantics is a concept that extracts information from the attributes of an image to form a process of transferring, mapping, and fusion of low-level information to high-level semantics to describe or express the original image^21^.

Many literatures on image semantics only use one of the underlying features such as color, texture or shape, which is still very limited. Here, the semantic segmentation is chosen to cover the four semantic features of color, texture, shape and size of remote sensing images, which greatly improves the robustness of semantic feature extraction of remote sensing images.

Semantic feature extraction of land cover remote sensing image images segmented in “Semantic segmentation algorithm for land cover remote sensing images based on deep inverse convolutional neural network” section using an improved sequential clustering algorithm, setting the dimensions of $O_{j}$ is $\phi {*}\varphi$, for which interest pixel clustering is performed. An important influencing factor here is the selection of thresholds, which requires manual intervention. Based on the a priori knowledge, the 2 thresholds for each target object hue in the segmented land remote sensing multi-target semantic image are specified as $k_{j1}$, $k_{j2}$, the saturation threshold is $r_{j}$, for the fulfillment of $k_{j1} < k_{h} < k_{j2}$ and $r_{h} < r_{j}$ of pixel clustering, here $k_{h}$, $r_{h}$ are Hue and saturation components of pixel $O_{j}$. The process of clustering is a step-by-step refinement process, i.e., the threshold is continuously adjusted according to the subjective judgment of the clustering results, and this method is similar to the process of human cognition. After clustering, the semantic features $L_{1} ,L_{2} ,\ldots,L_{M}$ of the target object in the image are in total $M$. In this study, the $M{ = }4$.

Color is the most direct semantic feature that describes the content of an image, and it is recognized that color is more stable. In response to $L_{1} ,L_{2} ,\ldots,L_{M}$, set the total number of pixels of each target object to be $N_{1} ,N_{3} ,\ldots,N_{M}$, respectively. The average hue semantic values for each target object is:

7 $$K_{avg - j} = \frac{1}{{N_{j} }}\sum\limits_{n = 1}^{{N_{j} }} {K_{j.n} }$$

Among them, $K_{j.n}$ is the tone semantic value of the $j$th pixel coordinates of the $j$th target object.

Texture is a property specific to all physically existing surfaces. In order to utilize the spatial information about the relative positions of pixels, the description of symbiotic matrices is used. Let $\overline{o}$ be the set of pairs of pixels $\left( {o_{1} ,o_{2} } \right)$ with specific spatial associations in the target region $O$, define the angular second-order moments $asm$ based on the covariance matrix is:

8 $$asm = \sum\limits_{{o_{1} }} {\sum\limits_{{o_{2} }} {K_{avg - j} \left( {Q\left( {o_{1} ,o_{2} } \right)} \right)^{2} } }$$

Among them, $Q$ is the gray value.

This feature is a measure of the smoothness of the segmented image, the less smooth the region is, the more the image is smoothed. The more homogeneous for $Q\left( {o_{1} ,o_{2} } \right)$, the smaller the value of the angular second-order moments $asm$.

When the boundaries of a target object are known, it is easiest to use the dimensions of its outer rectangle to characterize its basic shape and size.

There are countless external rectangles of a target object, and the real reflection of the shape and size characteristics of the target object is the minimum area of the external rectangle of the target object. Rotate the target object within 90° at equal intervals, record the parameters of the external rectangle in the direction of each coordinate system, take the rectangle with the smallest area as the length and width in the sense of the main axis, and then the length-to-width ratio of the rectangle, the length-to-width ratio of the rectangle, and the length-to-width ratio of the rectangle $C/P$ as a semantic parameter for shape features. The smaller the angle of rotation, the more accurate the result. However, it will bring about a reduction in efficiency. Considering the two factors, we choose the equal interval rotation angle of 6°, and get the following.

9 $$\left( \frac{C}{P} \right)_{j} = \min \left( \frac{C}{P} \right)_{1j} , \ldots ,\left( \frac{C}{P} \right)_{16j}$$

**Figure 4:**
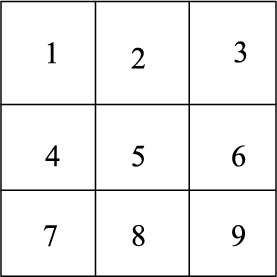
Division of object positions in images.

$O$ is divided into nine regions, as shown in Fig. 4.

The area where each target object is located is determined according to the coordinates of its center of gravity.

### Random forestalgorithm based semantic feature classification and recognition method for remote sensing images

Taking the semantic features $L_{1} ,L_{2} ,\ldots,L_{M}$ extracted in “Semantic feature extraction method for remote sensing images based on improved sequential clustering algorithm” section as the recognition object of remote sensing image semantic feature classification and recognition method based on random forest algorithm, random forest (RF) is a classification algorithm with high accuracy, which can be used to process large quantities of input data, and has high computing efficiency and speed. At present, it is widely used in various fields.

RF uses the CART decision tree as the basic learner for integrated learning. The decision tree is a tree data structure composed of root nodes, intermediate nodes and leaf nodes. Using Bagging algorithm, from training set $L^{\prime}$ randomly acquired $M$ training subset $L^{\prime} = \left\{ {L^{\prime}_{1} ,L^{\prime}_{2} , \ldots ,L^{\prime}_{M} } \right\}$ of independent and identically distributed, and construct generative correspondences based on different training subsets $M$ different decision trees $\sigma = \left\{ {\sigma_{1} ,\sigma_{2} , \ldots ,\sigma_{M} } \right\}$. CART decision tree passes $Gini$ coefficients as criteria for node feature selection, if there is $M$ species-specific instance in the sample set $L^{\prime}$, the coefficients $Gini$ is calculated as follows:

10 $$Gini\left( {L^{\prime}} \right) = 1 - \sum {\left[ {\lambda \left( j \right)*\lambda \left( j \right)} \right]}$$

Among them. $\lambda \left( j \right)$ is the proportion of semantic feature samples of class $j$ in the dataset on the current node, when $L^{\prime}$ divided into $L^{\prime}_{1}$, $L^{\prime}_{2}$ two subsets of semantic feature samples, thecoefficients $Gini$ is defined as follow:

11 $$Gini = Gini\left( {L^{\prime}} \right) - Gini\left( {L^{\prime}_{1} } \right) - Gini\left( {L^{\prime}_{2} } \right)$$

Select the attribute that minimized the $Gini$ coefficient as the split attribute of the node, and the node threshold is set, satisfying the criteria for stopping splitting. For the $j$th CART decision tree, which trains the subset of semantic feature samples from the root node. If the termination condition is met, the current node is set as the leaf node; If the termination conditions are not met, use $Gini$ coefficient selection $L^{\prime}$ for an optimal semantic feature, divide the semantic feature samples on the current node into left and right sub nodes, and continue to train other nodes until all nodes have been trained or marked as leaf nodes. After all CART decision trees are trained, each tree can predict the test sample set according to the node threshold, and vote to determine the final classification result of the entire random forest based on the classification results of each tree. Finally, the semantic classification is completed by inputting the test sample of the semantic characteristics of land cover remote sensing image into formula (12).

12 $$\Omega = \arg \mathop {\max }\limits_{\eta } \sum\limits_{j = 1}^{M} {GiniJ\left( {L^{\prime}_{M} = \eta } \right)}$$

Among them, $\Omega$ denotes the voting result for semantic feature classification; the $\eta$ is a semantic feature type for land cover remote sensing images; the $J$ is a schematic function.

## Experimental analysis

### Experimental design

This study selected farmland in Siping City, Jilin Province, China as the research area. The land cover types in this area mainly include crops, forests, grasslands, and construction land. In order to obtain high-quality surface information, high-resolution Jilin-1 satellite is used to obtain satellite images. The satellite image was obtained on June 15, 2023 at 10:00 am, with a spatial resolution of 30 cm. The satellite image is open-source data provided by Changguang Satellite Technology Co., Ltd (https://www.jl1mall.com/lab/).

During the training of deep deconvolution neural networks, careful adjustments were made to the hyperparameters. By trying different combinations of parameters such as learning rate, batch size, and iteration number, the classification performance of the model is optimal when the learning rate is set to 0.001, batch size is 32, and iteration number is 100.

In order to comprehensively evaluate the performance of the proposed method, multiple evaluation metrics were used. Specifically, the intersection to union ratio and F1 score were calculated to evaluate the classification results. By counting the number of true cases (TP), false positive cases (FP), true negative cases (TN), and false negative cases (FN), the specific values of each indicator can be obtained to further evaluate the performance of the model. Meanwhile, to ensure the generalization ability and robustness of the model, representative satellite image datasets were used for training and validation. This dataset contains satellite images from different regions and seasons to ensure the diversity of the dataset. The dataset was randomly divided, with 70% of the data used for training, 20% for validation, and the remaining 10% for testing.

### Analysis of the training effect of deep inverse convolutional neural networks

**Figure 5:**
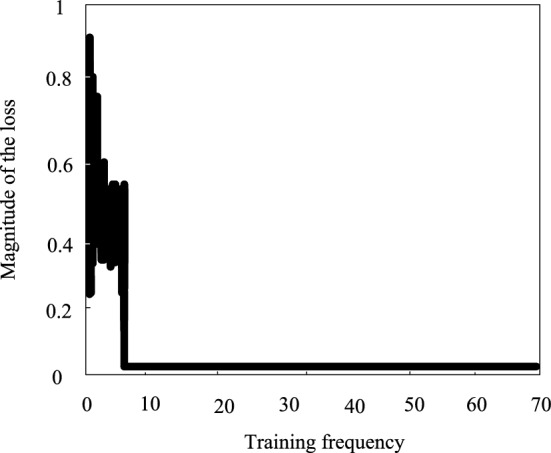
Training loss value of deep deconvolution neural network.

**Figure 6:**
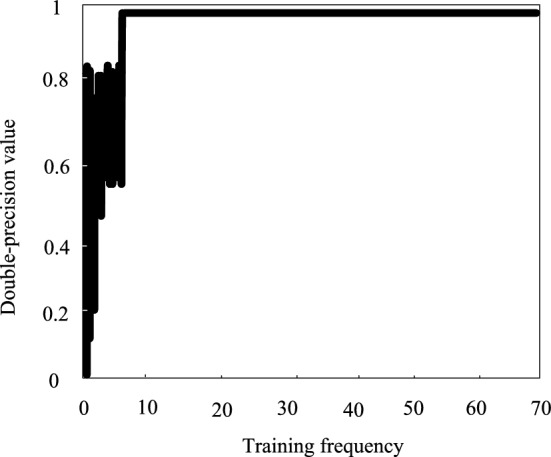
Training accuracy value of deep deconvolution neural network.

Before analyzing the effect of this method on the semantic segmentation of land cover remote sensing images, the training effect of the deep inverse convolutional neural network used in this method is tested. Figures 5 and 6 show the training loss and accuracy curves of the dataset, with the increase of training rounds, the curves of the loss and accuracy values tend to stabilize within 10 training rounds, which indicates that the method has good training accuracy, and the specific training results are shown in Table 1.

**Table 1:** Training effectiveness.

Data set	Training set	Test set
Magnitude of the loss	0.0421	0.0321
Double-precision value	0.9898	0.9973

As the data in Table 1 show, the training effect of deep inverse convolutional neural network is ideal, and the loss values of both the training set and the test set are low, and the accuracy value is more than 0.98, which can be used in the problem of semantic segmentation of land remote sensing images.

### Analysis of the effect of semantic segmentation of land cover remote sensing images

**Figure 7:**
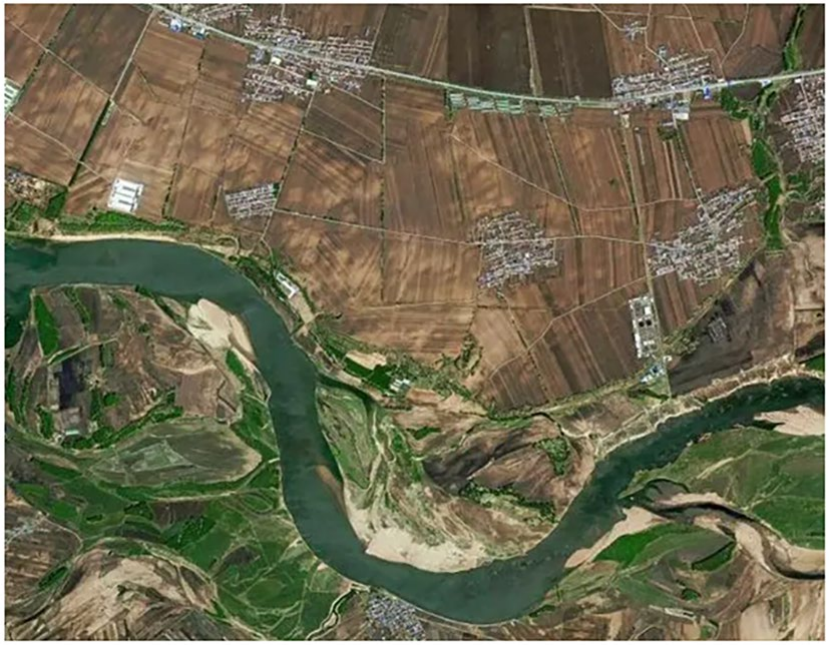
Original land remote sensing images.

**Figure 8:**
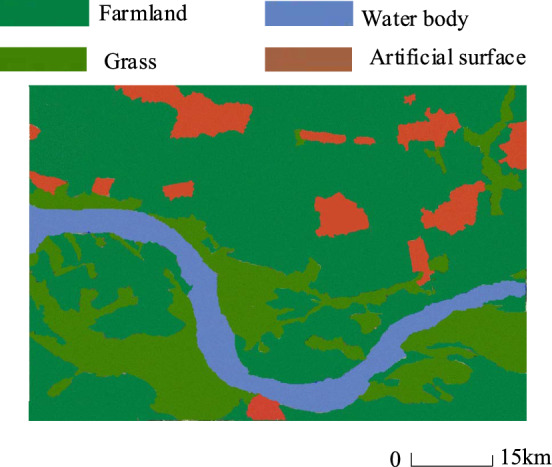
The semantic segmentation effect of land remote sensing images using the method described in this paper.

Firstly, we test the semantic segmentation effect of this paper’s method on the land remote sensing image collected by UAV, Fig. 7 is the land remote sensing image before segmentation, combined with the actual image target semantic type information, Fig. 8 is the semantic segmentation effect of the land remote sensing image after this paper’s method utilizes the deep inverse convolutional neural network.

As shown in Fig. 8, the semantic segmentation contours of different targets in the semantic segmentation effect map of land remote sensing image are obvious after segmentation by the method of this paper using deep inverse convolutional neural network. There are obvious contour demarcation lines between farmland, grassland, water bodies and artificial land surface.

The experiment uses two parameters to quantitatively evaluate the effect of this method on remote sensing image semantic segmentation, namely Dice similarity coefficient and Hausdorff distance. Dice similarity coefficient calculates the similarity of two target semantic contour regions. Set the point set included in the 2 target semantic contour areas as $o_{1}$, $o_{2}$, which is defined as:

13 $$Dice\left( {o_{1} ,o_{2} } \right) = \frac{{2\left| {o_{1} \cap o_{2} } \right|}}{{\left| {o_{1} } \right| + \left| {o_{2} } \right|}}$$

The Hausdorff distance reflects the maximum difference between the two target semantic contour point sets, which is defined as:

14 $$Hausdorff\left( {o_{1} ,o_{2} } \right) = \max \left( {\tau \left( {o_{1} ,o_{2} } \right),\tau \left( {o_{2} ,o_{1} } \right)} \right)$$

Among them, $\tau$ represents the one-way Hausdorff distance from the target semantic contour point set. The smaller the Hausdorff distance, the higher the segmentation accuracy.

**Figure 9:**
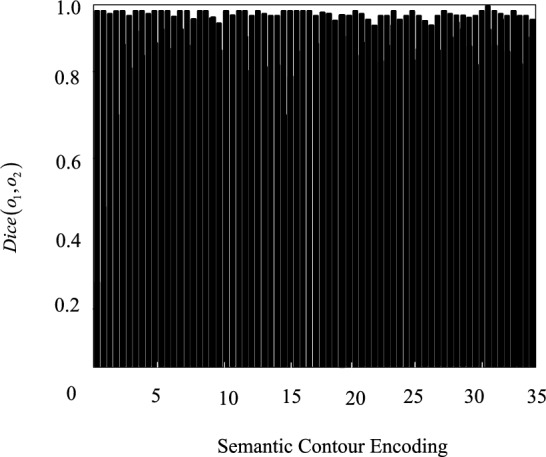
Dice similarity coefficient calculation results.

**Figure 10:**
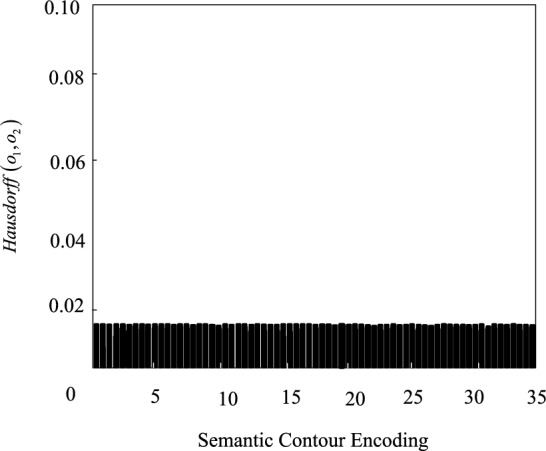
Hausdorff distance calculation result.

Figures 9 and 10 show the test results of Dice similarity coefficient and Hausdorff distance.

It can be seen from Figs. 9 and 10 that the segmentation accuracy of the method in this paper is close to the ideal state in Dice similarity coefficient and Hausdorff distance, which meets the semantic segmentation requirements of actual land remote sensing images.

### Analysis of the effect of semantic classification of land cover remote sensing images

An effective accuracy evaluation system is an important guarantee for evaluating the semantic classification results of land cover remote sensing images by the method in this paper, and it is also the basis for the application of data products. The intersection and concatenation ratio (ICR), which is commonly used in deep learning, is used to evaluate the accuracy of the method ($\zeta_{2}$) and F1 scores ($\zeta_{3}$) these 2 metrics to evaluate the single-class classification accuracy of the semantic classification results of remote sensing images, calculated as shown in Eqs. (15) and (16), respectively.

15 $$\zeta_{2} { = }\frac{TP}{{TP + FP + FN}}$$

16 $$\zeta_{3} { = 2} \times \frac{\chi \times \rho }{{\chi + \rho }}$$

Among them, $\zeta_{1}$ denotes the overall pixel accuracy; the $\zeta_{2}$ denotes the ratio of intersection and merger. $\zeta_{3}$ represents the semantic classification results of remote sensing images for F1 score; $TP$, $TN$ denote the number of pixels in which positive samples are accurately categorized in the semantic classification results of remote sensing images, and the number of pixels in which negative samples are correctly categorized in the classification results, respectively. $FP$, $FN$ denote the number of pixels in which positive samples are misclassified in the classification result, and the number of pixels in which negative samples are misclassified in the classification result, respectively; and $\chi$, $\rho$ denote checkout rate and recall rate, the former represents the proportion of positive samples categorized as positive cases, while the latter represents the proportion of positive cases categorized to the total number of positive cases, respectively.

In order to verify the classification performance of the proposed deep learning method on different date images, further accuracy estimation was conducted. Specifically, three other remote sensing images with similar features to the original dataset (June 18th, June 22nd, and June 25th) were selected, and the proposed method was applied for semantic classification of land cover. If the semantic classification targets for remote sensing images are forest land, grassland, water body, cultivated land, and artificial surface, the semantic classification results of this method for land remote sensing images are shown in Table 2.

**Table 2:** Semantic classification results of land remote sensing images using the method proposed in thispaper.

Date	Test indicators	Woodland	Grass	Water body	Cultivated land	Artificial surface	Mean value
June 18th	$\zeta_{2}$	0.9876	0.9879	0.9879	0.9876	0.9875	0.9877
	$\zeta_{3}$	0.9898	0.9898	0.9899	0.9875	0.9987	0.9911
June 22th	$\zeta_{2}$	0.9976	0.9956	0.9864	0.9756	0.9865	0.9883
	$\zeta_{3}$	0.9976	0.9966	0.9943	0.9797	0.9975	0.9831
June 25th	$\zeta_{2}$	0.9943	0.9865	0.9876	0.9866	0.9854	0.9841
	$\zeta_{3}$	0.9943	0.9806	0.9876	0.9888	0.9866	0.9856

According to the experimental results in Table 2, the deep learning method demonstrated high accuracy in land cover classification on land remote sensing images of different dates. On the image of June 18th, our method showed high $\zeta_{2}$ and $\zeta_{3}$ in the classification of forest land, grassland, water body, cultivated land, and artificial surface, both exceeding 0.98. This indicates that the method proposed in this paper can accurately identify various types of land cover. On the image of June 22nd, our method continued to demonstrate high classification accuracy, especially achieving 0.9976 $\zeta_{2}$ and 0.9976 $\zeta_{3}$ on forest land, demonstrating very accurate classification results. In other categories, $\zeta_{2}$ and $\zeta_{3}$ are relatively low but still maintain a high level. On the image of June 25th, our method once again demonstrated high classification accuracy, with both $\zeta_{2}$ and $\zeta_{3}$ exceeding 0.98, verifying the robustness and stability of the method. Overall, the deep learning method proposed in this paper has achieved significant classification results on land remote sensing images of different dates.

**Figure 11:**
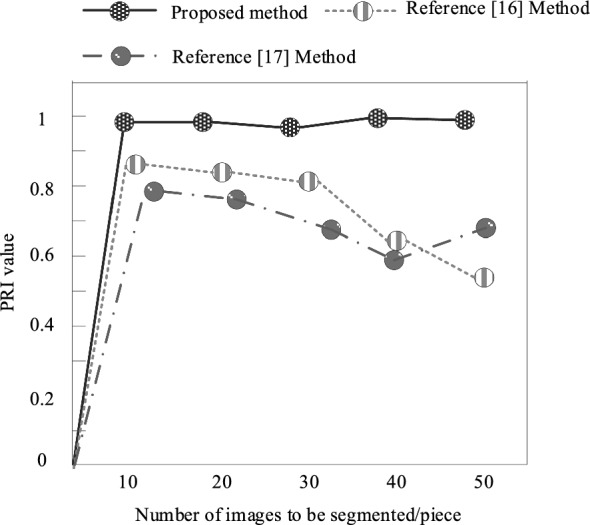
Test results of image segmentation performance using different methods.

To further validate the effectiveness of the proposed method, the Probability Rand Coefficient (PRI) was selected as the evaluation metric. PRI is an indicator that calculates the consistency between image segmentation results and manual segmentation reference maps through statistical calculations. Its value range is usually between [0,1], with a value close to 1 indicating good segmentation performance. The experiment tested the image segmentation performance of different methods and calculated their respective PRI values. The segmentation performance of the proposed method was compared with the methods in references^15,16^. The test results of the image segmentation performance of different methods are shown in Fig. 11.

Analyzing the experimental results in Fig. 11, it can be seen that the proposed method obtained a PRI index close to 1 during segmentation performance testing, indicating good segmentation performance. The PRI index of the methods in reference^15^ and reference^16^ are both below 0.9. Through comparison, it can be seen that the proposed method has significantly better test results than these two methods. It can be concluded that the proposed method performs better in image segmentation.

### Analysis of semantic classification time for land cover remote sensing images

In order to evaluate the time consumption of semantic classification of land cover remote sensing images, the following experiments were conducted and the time required for different methods to process image data of the same scale was recorded. A set of remote sensing images of land cover with different resolutions and sizes was selected as the input dataset, including different types of landforms such as urban areas, farmland, and forests. In the experiment, the proposed method, methods from references^15,16^ were used for testing, and the experimental results are shown in Table 3.

**Table 3:** Time consumption results of semantic classification of land cover remote sensing images using different methods.

Number of images/frame	Segmentation time/ms		
	Proposed method	Reference^15^ method	Reference^16^ method
100	10	15	18
500	40	55	65
1000	80	110	130

According to experimental analysis, the proposed method exhibits lower average time consumption, and as the number of images increases, the time consumption of all methods also shows an increasing trend. Compared to the methods in references^15,16^, the proposed method has higher computational efficiency and performs well in semantic classification tasks of large-scale image data. Therefore, the proposed method can quickly and effectively process land cover remote sensing images, and has broad application prospects.

## Discussion

To confirm the robustness of the proposed deep learning model, the following measures were taken. Firstly, select remote sensing images of different dates with similar features to cover changes in different seasons and environmental conditions. This can verify the classification performance of the model on images with different dates. Secondly, using the same deep learning methods and classification objectives, classify images on different dates. By comparing the classification results, the performance differences of the model can be evaluated on different dates. Finally, the intersection to union ratio and F1 score are used as evaluation metrics to measure the classification accuracy of the model on each category. By comparing classification metrics under different dates, the robustness and stability of the model can be evaluated.

In summary, the robustness of the proposed deep learning model on different date images can be confirmed through the above measures. This experimental design can help verify the classification accuracy of the model under different seasons and environmental conditions, and determine its accuracy and stability under changing conditions.

## Conclusion

This paper proposes a semantic segmentation and classification method for land cover remote sensing images based on deep deconvolution neural networks. Through experimental verification, this method has shown superior performance in remote sensing image semantic segmentation and classification tasks. Specifically, the deep deconvolution neural network trained on the dataset by this method achieved ideal values in both training loss and accuracy, indicating that this method has good training performance. In practical applications, this method performs semantic segmentation on land remote sensing images collected by drones, and the results show that the semantic segmentation contours of different targets are obvious, meeting practical needs. In addition, the land cover classification accuracy of this method on different date images is significantly higher than other methods, further proving the effectiveness and stability of its classification performance. Finally, through comparative experiments with other methods, the proposed method performs better in image segmentation and classification, with lower computational time and higher computational efficiency, and shows good performance in semantic classification tasks of large-scale image data. In summary, the method proposed in this paper has broad application prospects in semantic segmentation and classification of land cover remote sensing images.

## Data Availability

The datasets used and/or analyzed during the current study available from the corresponding author on reasonable request.
